# Objective and Subjective Dementia Caregiving Burden: The Moderating Role of Immanent Justice Reasoning and Social Support

**DOI:** 10.3390/ijerph17020455

**Published:** 2020-01-10

**Authors:** Yanchun Cao, Fan Yang

**Affiliations:** 1 Faculty of Economics and Management, East China Normal University, Shanghai 200062, China; yccao@sem.ecnu.edu.cn; 2 School of International and Public Affairs, China Institute for Urban Governance, Shanghai Jiao Tong University, Shanghai 200030, China

**Keywords:** family dementia caregiving, objective burden, subjective burden, immanent justice reasoning, social support

## Abstract

Caregiving burden significantly effects the physical and mental health of family dementia caregivers. While the association between objective caregiving burden (OCB) and subjective caregiving burden (SCB) of family dementia caregivers is well documented, little is known as with how the association is moderated by the configuration of intrapersonal resource (e.g., immanent justice reasoning) and interpersonal resource (e.g., social support). The present study collected cross-sectional data on 157 major family caregivers of non-institutionalized persons with dementia in an urbanizing region of Western China’s Sichuan Province. They responded to questions on daily time spent on caregiving, the short version of Zarit Burden Interview (ZBI), a sub-scale of a caregiver meaning scale, Social Support Rating Scale (SSRS), and demographic questions. Controlling for the demographic variables of the caregivers, this study found that the objective and subjective dementia caregiving burden were significantly associated (*p* < 0.001), and immanent justice reasoning was positively correlated with subjective burden (*p* < 0.01). Moreover, the association between OCB and SCB was significantly positive when social support and immanent justice reasoning were both high (*p* < 0.001), but neutral when social support was high and immanent justice reasoning was low. The association between OCB and SCB was significantly positive when social support and immanent justice reasoning were both low (*p* < 0.05), but neutral when social support was low and immanent justice reasoning was high. This research suggests the importance of developing intervention programs that consider the configuration of the external supporting resources and internal meaning-making of caregiving of the family dementia caregivers.

## 1. Introduction

The physical and mental health effects of caregiving burden on the caregivers in turn causes harm to the quality of life of the persons with dementia [1,2]. The problem could be an even tougher challenge in developing countries. Over 90% of people with dementia in the developing world were cared for at home, mainly due to the underdevelopment of elderly care institution and the prevalence of traditional family values [3]. It is also worth noting that the number of people with dementia will increase from 35.6 million in 2010 to 66 million in 2030, and the main increase will take place in low-income and middle-income countries [4,5]. In China, the population living with dementia is estimated to increase from 9.6 million in 2010 to 23.3 million in 2030, with a higher prevalence rate in the rural sector where family caregiving prevails [5]. Therefore, researching into dementia caregiving burden of family caregivers in developing countries may have significant policy implication.

Caregiving burden refers to the stress, tension, and anxiety that caregivers experience when they are faced with challenges when caring for their care receiver [6]. Past literature suggests that caregiving burden should be classified as objective burden and subjective burden, as factors contributing to and the impact of these types of burden on caregivers’ physical and mental health can differ [7]. Objective burden refers to the inputs relevant with caregiving activities, which could be measured by time spent on caregiving and care recipients’ functional level. Subjective burden focuses on the self-perceived impact of the objective burden on the caregivers [8]. Many studies have documented the significant association between objective and subjective caregiving burden of family dementia caregivers. That is, higher objective burden is correlated with increased subjective burden [9,10].

At the same time, an increasing body of literature argues that dementia caregivers under higher objective burden are not necessarily feeling higher subjective burden, mainly due to the buffering role of intrapersonal and interpersonal resources for the caregivers [11,12,13]. In dementia caregiving, interpersonal resources refer to the instrumental and psychological supports from the state, community, and from family members [14]. As a type of interpersonal resource, social support’s buffering effect on dementia caregivers’ subjective burden has been well documented [15,16]. Informal dementia care imposed heavy financial burden on families, and material supports could be of critical importance in tackling dementia caregiving burden for families in developing countries [17]. Moreover, welfare service provision from the state, NGOs, as well as the instrumental and psychological supports from friends and family members could play a positive role in dealing with dementia caregiving burden [16,18].

On the other hand, intrapersonal resources mainly refer to one’s personality and the social norms, family values, and outlooks on life internalized in a specific cultural context [19]. These resources may decide the caregivers’ obligations towards family members with dementia and the degree they make meaning of the dementia caregiving process [20]. Immanent justice reasoning could be one’s intrapersonal resources when dealing with life challenges [21].

Immanent justice reasoning derives from sense of fairness and involves causally attributing a deserved outcome to someone’s prior moral deeds or character, even when such a causal connection is physically implausible [22]. That is, the sense of fairness construes a misfortune as a way to compensate for a misdeed, while good intent and deeds contribute to future good fortune. In its long history, Chinese culture nurtures the belief in immanent justice with the theory of nature and fate in Confucianism and the ideas of reincarnation in Buddhism and Taoism [23]. The thoughts of retribution for sin, compensation for debt in a previous life, and merits accumulation for afterlife has been an important spiritual basis for Chinese people [24]. These thoughts still have profound influences in contemporary Chinese society, especially in the rural sector [25]. According to the just-world theory, people rely more on immanent justice reasoning to make causal attributions when systematic thought is interrupted [26]. Therefore, immanent justice reasoning could constitute a protective resource for people to rely on when personal control is lacking. However, its role in shaping the perception and motivation of family caregiving is rarely studied, and an exploration of this culture-related intrapersonal factor could contribute to the improvements of caregiving burden intervention programs designed and implemented in the local context.

It is also worth noting that although much research has been done as with the effects of either intrapersonal factors or interpersonal factors on caregiving burden, little literature has still been on how they jointly shape dementia caregivers’ subjective burden. The present study aimed to fill this gap by examining how the configuration of immanent justice reasoning and social support shaped the relationship between the objective caregiving burden and the subjective caregiving burden of family dementia caregivers in rural China.

## 2. Materials and Methods

The present study utilized the quantitative data collected through the questionnaire survey on family caregivers of persons with dementia. Doctors from the local mental health hospital, with the assistantship of the community healthcare center staff, would pay regular visits to households with person with dementia. They were invited to do the face-to-face interviews in one normal visit.

### 2.1. Participants and Procedures

The present study was based on the responses of 157 major family caregivers of persons with dementia in Xinjin County, Western China’s Sichuan Province. After retrieving the medical records from the local mental health hospital and the statistics of a countywide epidemiological survey on mental health, we found that the county had a total of 377 non-institutionalized people who were diagnosed with dementia till October of 2016. Between January and February of 2018, the research team contacted the families of these persons with dementia and successfully completed interviews with the 157 caregivers who had cared for the family member with dementia for one year or longer. The persons with dementia in the 157 households were aged between 47 and 97 years (mean = 77.58 years, *SD* = 10.30), with 64 males and 93 females. Other families were not interviewed due to the following reasons: 152 people with dementia passed away, 37 were transferred to be cared for in an institution, 24 were relocated from the original residence and were out of touch, five did not have a family caregiver, and two people with dementia were cared for by the current family caregiver for less than one year.

Doctors in the local mental health hospital were recruited and trained to conduct face-to-face interviews. Written informed consent was obtained from all the participants (except for the illiterate, who only provided oral consent) before beginning the data collection. The project received approval from the Human Research Ethics Committee of the corresponding author’s university.

### 2.2. Measures

#### 2.2.1. Subjective Caregiving Burden

The subjective burden of the dementia caregiver was measured by a short version of the Zarit Burden Interview (ZBI), whose validity and reliability have been confirmed [27]. The scale comprises of 12 questions graded on a scale from 0 to 4, according to the presence or intensity of an affirmative response, and measures the caregiver’s health, psychological wellbeing, social life, finances, and the relationship between the caregiver and person with dementia. It includes questions such as “Do you feel that because of the time you spend with your relative that you don’t have enough time for yourself?”, “Do you feel angry when you are around your relative?”, “Do you feel your health has suffered because of your involvement with your relative?”. In the present study, the Cronbach’s α of the scale was 0.91.

#### 2.2.2. Objective Caregiving Burden

Objective caregiving burden was measured by a self-report of caregivers regarding the daily hours of caregiving. The caregivers selected from one of the five items, namely, “less than 1 h” = 1, “1–4 h” = 2, “4–7 h” = 3, “7–10 h” = 4, and “above 10 h” = 5.

#### 2.2.3. Immanent Justice Reasoning

The present study used a sub-scale of a caregiver, meaning a scale developed and validated in the Chinese culture context to measure family dementia caregivers’ immanent justice reasoning [28]. The sub-scale focused on the factor of belief in karma in the caregiving process. It is comprised of five items, namely, “Caregiving is compensation to my ill family member”, “I believe I owe my ill family member from a previous life, so I need to repay the debt by caregiving”, “I think it is a punishment by a god, so I need to assume the task of caregiving”, “It is my karma that determined the situation, so I need to assume the task of caregiving”, “I need to give care to have a clear conscience”. The response for each item was rated with a five-point scale, and the Cronbach’s α of the scale was 0.79.

#### 2.2.4. Social Support

The Social Support Rating Scale (SSRS), developed to cater for China’s context, has sub-scales that measure objective support, subjective support, and support utilization [29]. This 10-item scale has been widely used and has demonstrated good reliability for different populations in China [30]. Higher scores indicate greater social support for the respondents. In the present study, the Cronbach’s α coefficient was 0.84 for the SSRS.

#### 2.2.5. Control Variables

The present study controlled for the family caregivers’ gender, age, educational level, and income. Caregivers’ gender was measured with male = 0, female = 1. Age was measured in years. Educational level was measured with illiterate = 0, elementary school = 1, junior high school or equivalent = 2, high school or equivalent = 3, college/university or above = 4. Income measured the caregivers’ self-reported monthly income (lower than 1000 yuan = 1, 1000–3000 yuan = 2, 3001 yuan or above = 3).

### 2.3. Analytical Plan

First, descriptive statistics of the research variables were presented, which was followed by zero-order correlations between the variables used in the regression analyses. Then, hierarchical moderated regression analysis was performed to test the research hypotheses. Hierarchical regression analysis allows for a comparison between alternative models with and without interaction terms, where an interaction effect only exists if the interaction term contributes significantly to the variance explained in the dependent variable over the main effects of the independent variables [31]. In the present study, it includes four models. Model 1 tested the effects of the control variables. Model 2 tested the main effects of OCB, immanent justice reasoning, and social support. Model 3 tested the effects of two-way interactions, namely OCB—immanent justice reasoning interaction, OCB—social support interaction, and immanent justice reasoning—social support interaction. Model 4 tested the effects of the interaction term of OCB, immanent justice reasoning, and social support. The independent variables and the proposed moderators were mean-centered before testing the interactions [32]. Also, Kolmogorov-Smirnov test was conducted to check for normality, which supported the univariate normality assumption (*p* > 0.05). In addition, the study assessed the variance inflation factor (VIF) values. It is found that the VIF values for research variables were between 1.09 and 1.91, indicating that no significant multicollinearity problems existed. IBM SPSS with PROCESS Macro was utilized to do bootstrap analyses in the present study. The data obtained from 10,000 bootstrap samples were used [33].

## 3. Results

### 3.1. Participant Characteristics

Table 1 presents descriptive statistics of the research variables. 51.0% of the family caregivers were females. The mean age of them was 58.54 years (Range = 27–89 years, *SD* = 13.72), and 42.6% of them were aged 60 and above. Most of them had a low educational level, that is, 53.5% did not receive junior high school education. About one third of them had a monthly income lower than 1000 yuan.

Table 2 presents zero-order correlations between the variables used in the regression analyses. Significant positive correlation existed between the OCB and SCB (*r* = 0.38, *p* < 0.001). No significant association was found between the two moderators, namely immanent justice reasoning and social support (*r* = 0.11, *p* = 0.001).

### 3.2. Hierarchical Moderated Regression Analysis Results

Table 3 reports the results of the hierarchical regression analyses. At the first step, the study entered the control variables to form the Model 1. Then, in step 2, the main effects of OCB, immanent justice reasoning, and social support, together with the control variables were included into the Model 2, which explained a significant share of the variance in SCB (Model 2: R^2^ = 0.17, *p* < 0.001).

In step 3, the two-way interaction terms were entered to form the Model 3. Interestingly, this addition did not increase the explained variance in subjective caregiving burden significantly. The Model 3 did not support an independent moderating effect of immanent justice reasoning or social support. While in step 4, the study entered the three-way interaction term to test the configuration hypothesis. The addition of this product term significantly increased the variance explained in subjective caregiving burden (R^2^ = 0.23, *p* < 0.05). In Model 4, the interaction term between OCB and social support (β = 0.17, *p* < 0.05) and the interaction term of OCB, immanent justice reasoning, and social support (β = 0.23, *p* < 0.05) were found to explain variances in SCB significantly. It is also worth noting that immanent justice reasoning had a significant positive association with SCB (Model 4: β= 0.26, *p* < 0.01), and in Model 2–4, OCB and SCB were significantly associated (*p* < 0.001).

### 3.3. The Moderating Effect of the Configuration of Immanent Justice Reasoning and Social Support

**Figure 1:**
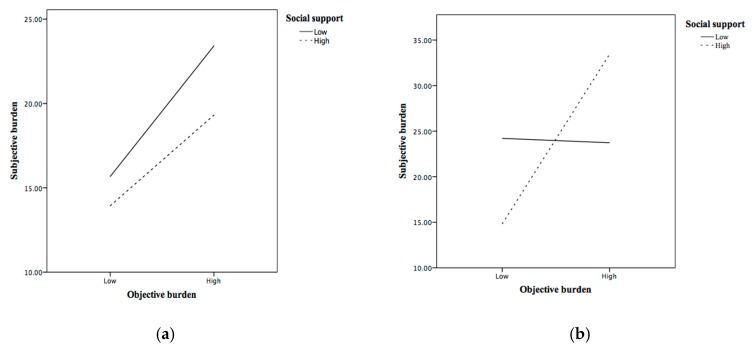
Moderating effects of immanent justice reasoning and social support on the relationship between objective and objective caregiving burden. (**a**) Low immanent justice reasoning; (**b**) High immanent justice reasoning.

To advance further interpretations, the study plotted these interaction effects for two levels of social support according to the pick-a-point approach [34]. The study defined the low level as minus one standard deviation from the mean and the high level as plus one standard deviation from the mean. For each level of immanent justice reasoning, the study plotted the association between OCB and SCB for low and high levels of social support. The results are shown in Figure 1. A simple slope analysis was performed for each regression line to test whether its slope was significantly different from zero. Figure 1a reveals that OCB had a significant and positive relationship with SCB among family dementia caregivers with low immanent justice reasoning and low social support (*b* = 3.11, *t* = 2.02, *p* < 0.05). Among caregivers with low immanent justice reasoning while high social support, OCB was unrelated to SCB (*p* = 0.23). Moreover, Figure 1b shows that the association between OCB and SCB was significantly positive when immanent justice reasoning and social support were both high (*b* = 7.48, *t* = 4.40, *p* < 0.001), but neutral when immanent justice reasoning was high and social support was low (*p* = 0.93).

## 4. Discussion

To better understand the influences of intrapersonal and interpersonal factors on caregiving burden of family dementia caregivers, the present study examined how the configuration of social support and immanent justice reasoning shaped the association between objective and subjective caregiving burden in rural China. The major findings include: (1) the subjective dementia caregiving burden was significantly associated with the objective dementia caregiving burden, (2) the focal association was significantly positive when the social support and immanent justice reasoning were both high, and (3) the focal association was significantly positive when the social support and immanent justice reasoning were both low.

In line with prior studies, this study demonstrated the positive relationship between OCB and SCB. For family dementia caregivers, longer caregiving time usually means more caregiving tasks, a limited social life, and more difficulties in maintaining work and life balance, which increase negative emotions that add to subjective burden [10,35,36]. Especially when caring family for members in the medium or advanced stages of dementia, the caregivers might have no or quite limited emotional interactions with the care recipients [37,38]. The hopelessness and guilt resulting from witnessing the fast functional decline of the beloved family member might exacerbate the caregivers’ subjective burden [39,40]. It is also worth noting that family caregivers in developing countries are faced with the scarcity of health services for people with dementia, which means heavier objective burden in the same caregiving period for them compared with their counterparts in developed regions [41]. This may contribute to the association between OCB and SCB in family dementia caregiving in the context of China.

The present study also reported that higher level of immanent justice reasoning was associated with the increased SCB among family dementia caregivers. Coping strategies like reappraisal of God’s powers and spiritual discontent were associated with negative perception of caregiving experience [42]. However, a more important reason could be that caregivers with high SCB were more likely to adopt immanent justice reasoning in order to justify the difficulties experienced in dementia caregiving [43].

One notable finding of the present study was the significant moderating effect of the configuration of social support and immanent justice reasoning on the association between OCB and SCB. It revealed the presence of two different moderating effects that remained hidden when social support was studied in isolation, which also explained the lack of support for an independent moderating effect of immanent justice reasoning or social support in the present study. Given a continuous increase in immanent justice reasoning, higher level of social support was initially associated with a weakened positive relationship between OCB and SCB, while at a certain inflection point, this moderating effect became that social support strengthens the focal positive association. These findings echoed the previous studies that demonstrate the interactions between intrapersonal and interpersonal resources could be significantly associated with the caregivers’ wellbeing [44,45,46]. Specifically, it appears that social support would be more useful in alleviating subjective burden for the family dementia caregivers with low immanent justice reasoning.

It is important to find that more social supports could lead to negative effects for family dementia caregivers with high immanent justice reasoning. The existing literature has rarely discussed the possible negative influence of social supports for vulnerable groups like family dementia caregivers [47]. Rather, social supports have been recognized as a panacea for improving mental health [48]. However, the willingness of the target group and the ways of delivering social supports are underexplored. Especially for family dementia caregivers who are faced with dementia stigma, receiving social and community supports means publicizing that they have a family member with dementia, which could be a significant life stressor in itself. The present study reveals the limitation of simply stressing the importance of social supports in family dementia caregiving, and the future study could further explore the roles of avoidance and stigma in order to give a fuller picture of the buffering effects of intrapersonal and interpersonal resources on the association between objective and subjective caregiving burden.

### 4.1. Study Limitations

The findings from this study should be interpreted with the following caveats. First, due to the cross-sectional nature of the data, this analysis was not able to determine the causality between the objective burden, social support, immanent justice reasoning, and the subjective burden of family dementia caregivers. Second, the measure of immanent justice reasoning was validated based on the Chinese population and it should be cautious when comparing the results with those from other cultural backgrounds, especially those with certain religions. Third, only daily hours of caregiving were utilized to measure the objective caregiving burden, while other variables, such as functional level and behavioral and psychological symptoms of dementia could also be good measures. Fourth, we used pick-a-point approach to further interpret the three-way interaction result. Though it is the most popular post hoc probing technique for three-way interactions, its limitations include that conditional values may not reflect the entire range of a continuous moderator variable and are sometimes chosen arbitrarily. Finally, this study was based in the context of rural China. Due to the variances in the level of secularization, education, and welfare sufficiency between rural and urban China, any generalization of the findings to the whole China, let alone to the world, should be cautious.

### 4.2. Implications for Practice

Despite the above limitations, the present study may have significant implications for dementia caregiving practice, especially for improving the wellbeing of family caregivers. Abundant evidence indicates that empowering family caregivers would increase the quality of life for both family caregivers and persons with dementia [49,50]. However, empowerment practices in existing schemes are largely on interpersonal aspect, such as building peer support network and dementia-friendly communities [51]. The present study reveals the importance of family caregivers’ intrapersonal resources and characteristics. That is, level of immanent justice reasoning should be considered when implementing interventions that add to the social supports for family caregivers of persons living with dementia. Interpersonal supports could be more effective for family caregivers with low level of immanent justice reasoning. While for family caregivers with high level of immanent justice reasoning, more interpersonal supports might lead to negative psychological outcomes.

## 5. Conclusions

By revealing the correlation between the configuration of objective burden, immanent justice reasoning, and social support with the subjective burden of family dementia caregivers in rural China, the present study enhanced the understanding of the moderating role of intrapersonal and interpersonal resources on the association between objective and subjective caregiving burden. The finding that immanent justice reasoning and social support had complementary effects on subjective burden of family dementia caregivers provided empirical support for contentions that both intrapersonal and interpersonal factors should be taken into account in designing and implementing intervention programs for family caregivers [46,52]. More specifically, caregivers’ immanent justice reasoning should be examined in order to guarantee the effectiveness of services on enhancing their social support.

## 6. Data Availability Statement

The data that support the findings of this study are available on request from the corresponding author. The data are not publicly available due to privacy or ethical restrictions.

## Figures and Tables

**Table 1:** Descriptive statistics of research variables (*N* = 157).

Variable	Range	*N* (%)/M (SD)
Gender		
Male		77 (49.0%)
Female		80 (51.0%)
Age (in years)	27–89	58.54 (13.72)
Education		
Illiterate		22 (14.0%)
Elementary school		62 (39.5%)
Junior high school or equivalent		54 (34.4%)
High school or equivalent		14 (8.9%)
College/university or above		5 (3.2%)
Income		
Lower than 1000 yuan		49 (31.2%)
1000–3000 yuan		57 (36.3%)
3001 yuan or above		51 (32.5%)
OCB ^1^	15	3.21 (1.22)
Immanent justice reasoning	0–20	8.34 (4.88)
Social support	0–46	29.80 (7.06)
SCB ^2^	0–45	20.76 (11.52)

Note: ^1^ OCB = objective caregiving burden; ^2^ SCB = subjective caregiving burden.

**Table 2:** Correlations of research variables (*N* = 157).

Variable	1	2	3	4	5	6	7
1. Gender							
2. Age	0.01						
3. Education	−0.07	−0.50 ***					
4. Income	0.03	−0.23 ***	0.44 ***				
5. OCB ^1^	0.07	0.23 ***	−0.09	−0.16 *			
6. Immanent justice reasoning	−0.01	−0.02	−0.11	0.04	−0.13		
7. Social support	0.14 ^†^	−0.38 ***	0.49 ***	0.34 ***	−0.19 *	0.11	
8. SCB ^2^	0.05	0.12	−0.10	−0.09	0.38 ***	0.13	−0.14 ^†^

Note: ^1^ OCB = objective caregiving burden; ^2^ SCB = subjective caregiving burden; ^†^ *p* < 0.10, * *p* < 0.05, *** *p* < 0.001.

**Table 3:** Results of hierarchical moderated regression analyses (*N* = 152).

Variables	Model 1	Model 2	Model 3	Model 4
Gender	0.05	0.04	0.05	0.06
Age	0.10	0.00	−0.02	0.01
Education	0.00	0.03	0.02	0.07
Income	−0.05	−0.00	0.10	0.01
OCB ^1^		0.38 ***	0.39 ***	0.34 ***
Immanent justice reasoning		0.17 *	0.19 *	0.26 **
Social support		−0.10	−0.10	−0.06
OCB * immanent justice reasoning			0.07	0.05
OCB * social support			0.11	0.17 *
Immanent justice reasoning * social support			0.00	0.08
OCB * immanent justice reasoning * social support				0.23 *
*R* ^2^	0.02	0.17	0.19	0.23
Adjusted *R*^2^	−0.01	0.13	0.14	0.16
*F* change	0.63	9.00 ***	1.14	5.53 *

Note: ^1^ OCB = objective caregiving burden; * *p* < 0.05, ** *p* < 0.01, *** *p* < 0.001.

## References

[1] Armstrong N.M., Gitlin L.N., Parisi J.M., Roth D.L., Gross A.L. (2018). Association of physical functioning of persons with dementia with caregiver burden and depression in dementia caregivers: An integrative data analysis. Aging Ment. Health.

[2] Terum T.M., Andersen J.R., Rongve A., Aarsland D., Svendsboe E.J., Testad I. (2017). The relationship of specific items on the Neuropsychiatric Inventory to caregiver burden in dementia: A systematic review. Int. J. Geriatr. Psychiatry.

[3] Prince M., Comas-Herrera A., Knapp M., Guerchet M., Karagiannidou M. (2016). World Alzheimer Report 2016: Improving Healthcare for People Living with Dementia: Coverage, Quality and Costs Now and in the Future.

[4] Wimo A., Guerchet M., Ali G.-C., Wu Y.-T., Prina A.M., Winblad B., Jönsson L., Liu Z., Prince M. (2017). The worldwide costs of dementia 2015 and comparisons with 2010. Alzheimer’s Dement..

[5] Xu J., Wang J., Wimo A., Fratiglioni L., Qiu C. (2017). The economic burden of dementia in China, 1990–2030: Implications for health policy. Bull. World Health Organ..

[6] Lai D.W. (2010). Filial piety, caregiving appraisal, and caregiving burden. Res. Aging.

[7] Wang J., Xiao L.D., He G.-P., Ullah S., De Bellis A. (2014). Factors contributing to caregiver burden in dementia in a country without formal caregiver support. Aging Ment. Health.

[8] Chou Y.-C., Fu L.-Y., Lin L.-C., Lee Y.-C. (2011). Predictors of subjective and objective caregiving burden in older female caregivers of adults with intellectual disabilities. Int. Psychogeriatr..

[9] Kim H., Chang M., Rose K., Kim S. (2012). Predictors of caregiver burden in caregivers of individuals with dementia. J. Adv. Nurs..

[10] Van der Lee J., Bakker T.J., Duivenvoorden H.J., Dröes R.-M. (2014). Multivariate models of subjective caregiver burden in dementia: A systematic review. Ageing Res. Rev..

[11] Montoro-Rodriguez J., Gallagher-Thompson D. (2009). The role of resources and appraisals in predicting burden among Latina and non-Hispanic white female caregivers: A test of an expanded socio-cultural model of stress and coping. Aging Ment. Health.

[12] Yang F., Ran M., Luo W. (2019). Depression of persons with dementia and family caregiver burden: Finding positives in caregiving as a moderator. Geriatr. Gerontol. Int..

[13] Yang F., Lou V.W.Q. (2016). Childhood adversities, urbanisation and depressive symptoms among middle-aged and older adults: Evidence from a national survey in China. Ageing Soc..

[14] Wang L.J., Zhong W.X., Ji X.D., Chen J. (2016). Depression, caregiver burden and social support among caregivers of retinoblastoma patients in China. Int. J. Nurs. Pract..

[15] Del-Pino-Casado R., Espinosa-Medina A., Lopez-Martinez C., Orgeta V. (2018). Sense of coherence, burden and mental health in caregiving: A systematic review and meta-analysis. J. Affect. Disord..

[16] Donnellan W.J., Bennett K.M., Soulsby L.K. (2017). Family close but friends closer: Exploring social support and resilience in older spousal dementia carers. Aging Ment. Health.

[17] Xu J., Zhang Y., Qiu C., Cheng F. (2017). Global and regional economic costs of dementia: A systematic review. Lancet.

[18] Stensletten K., Bruvik F., Espehaug B., Drageset J. (2016). Burden of care, social support, and sense of coherence in elderly caregivers living with individuals with symptoms of dementia. Dementia.

[19] Park C.L. (2010). Making sense of the meaning literature: An integrative review of meaning making and its effects on adjustment to stressful life events. Psychol. Bull..

[20] Quinn C., Clare L., Woods R.T. (2010). The impact of motivations and meanings on the wellbeing of caregivers of people with dementia: A systematic review. Int. Psychogeriatr..

[21] Baumard N., Chevallier C. (2012). What goes around comes around: The evolutionary roots of the belief in immanent justice. J. Cogn. Cult..

[22] Callan M.J., Sutton R.M., Harvey A.J., Dawtry R.J. (2014). Immanent justice reasoning: Theory, research, and current directions. Advances in Experimental Social Psychology.

[23] Smith A., MacEntee M.I., Beattie B.L., Brondani M., Bryant R., Graf P., Hornby K., Kobayashi K., Wong S.T. (2013). The influence of culture on the oral health-related beliefs and behaviours of elderly Chinese immigrants: A meta-synthesis of the literature. J. Cross-Cult. Gerontol..

[24] Yen C.-L. (2013). It is our destiny to die: The effects of mortality salience and culture-priming on fatalism and karma belief. Int. J. Psychol..

[25] Li Z. (2015). Between Tradition and Modernity: Philosophical Reflections on the Modernization of Chinese Culture.

[26] Callan M.J., Sutton R.M., Dovale C. (2010). When deserving translates into causing: The effect of cognitive load on immanent justice reasoning. J. Exp. Soc. Psychol..

[27] Bédard M., Molloy D.W., Squire L., Dubois S., Lever J.A., O’Donnell M. (2001). The Zarit Burden Interview: A new short version and screening version. Gerontology.

[28] Yen W.J., Ma W.F., Lu Y.C., Chang T., Lee S. (2011). The development and testing of a scale of Taiwanese caregiver meaning. J. Clin. Nurs..

[29] Xiao S. (1999). Social support rating scale. Mental Health Scale Manual, Quelling Updated Version.

[30] Deng J., Hu J., Wu W., Dong B., Wu H. (2010). Subjective well-being, social support, and age-related functioning among the very old in China. Int. J. Geriatr. Psychiatry.

[31] Jaccard J., Turrisi R., Jaccard J. (2003). Interaction Effects in Multiple Regression.

[32] Aiken L.S., West S.G., Reno R.R. (1991). Multiple Regression: Testing and Interpreting Interactions.

[33] Hayes A.F. (2013). Introduction to mediation, moderation, and conditional process analysis: A regression-based approach. J. Educ. Meas..

[34] Dawson J.F., Richter A.W. (2006). Probing three-way interactions in moderated multiple regression: Development and application of a slope difference test. J. Appl. Psychol..

[35] Del-Pino-Casado R., Frías-Osuna A., Palomino-Moral P.A., Pancorbo-Hidalgo P.L. (2011). Coping and subjective burden in caregivers of older relatives: A quantitative systematic review. J. Adv. Nurs..

[36] Xiao L.D., Wang J., He G.-P., De Bellis A., Verbeeck J., Kyriazopoulos H. (2014). Family caregiver challenges in dementia care in Australia and China: A critical perspective. BMC Geriatr..

[37] Egan M., Bérubé D., Racine G., Leonard C., Rochon E. (2010). Methods to enhance verbal communication between individuals with Alzheimer’s disease and their formal and informal caregivers: A systematic review. Int. J. Alzheimer’s Dis..

[38] Eggenberger E., Heimerl K., Bennett M.I. (2013). Communication skills training in dementia care: A systematic review of effectiveness, training content, and didactic methods in different care settings. Int. Psychogeriatr..

[39] Romero-Moreno R., Losada A., Marquez M., Laidlaw K., Fernández-Fernández V., Nogales-González C., López J. (2013). Leisure, gender, and kinship in dementia caregiving: Psychological vulnerability of caregiving daughters with feelings of guilt. J. Gerontol. Ser. B Psychol. Sci. Soc. Sci..

[40] Springate B.A., Tremont G. (2014). Dimensions of caregiver burden in dementia: Impact of demographic, mood, and care recipient variables. Am. J. Geriatr. Psychiatry.

[41] Jia L., Quan M., Fu Y., Zhao T., Li Y., Wei C., Tang Y., Qin Q., Wang F., Qiao Y. (2020). Dementia in China: Epidemiology, clinical management, and research advances. Lancet Neurol..

[42] Thombre A., Sherman A.C., Simonton S. (2010). Religious coping and posttraumatic growth among family caregivers of cancer patients in India. J. Psychosoc. Oncol..

[43] Callaby P., Coleman P.G., Mills M.A. (2012). Caregiving in Dementia: From Resentment to Forgiveness. J. Relig. Spiritual. Aging.

[44] Bekhet A.K., Elguenidi M., Zauszniewski J.A. (2011). The effects of positive cognitions on the relationship between alienation and resourcefulness in nursing students in Egypt. Issues Ment. Health Nurs..

[45] Bekhet A.K. (2013). Effects of positive cognitions and resourcefulness on caregiver burden among caregivers of persons with dementia. Int. J. Ment. Health Nurs..

[46] Hui L.O., Vaingankar J.A., Abdin E., Sambasivam R., Fauziana R., Tan M.E., Chong S.A., Goveas R.R., Chiam P.C., Subramaniam M. (2018). Resilience and burden in caregivers of older adults: Moderating and mediating effects of perceived social support. BMC Psychiatry.

[47] Maier C., Laumer S., Eckhardt A., Weitzel T. (2015). Giving too much social support: Social overload on social networking sites. Eur. J. Inf. Syst..

[48] Harada K., Sugisawa H., Sugihara Y., Yanagisawa S., Shimmei M. (2018). Social support, negative interactions, and mental health: Evidence of cross-domain buffering effects among older adults in Japan. Res. Aging.

[49] Hutchinson K., Roberts C., Daly M., Bulsara C., Kurrle S. (2016). Empowerment of young people who have a parent living with dementia: A social model perspective. Int. Psychogeriatr..

[50] Whitlatch C.J., Orsulic-Jeras S. (2018). Meeting the informational, educational, and psychosocial support needs of persons living with dementia and their family caregivers. Gerontol..

[51] Wiersma E.C., Denton A. (2016). From social network to safety net: Dementia-friendly communities in rural northern Ontario. Dementia.

[52] Dias R., Santos R.L., Sousa M.F., Nogueira M.M., Torres B., Belfort T., Dourado M.C. (2015). Resilience of caregivers of people with dementia: A systematic review of biological and psychosocial determinants. Trends Psychiatry Psychother..

